# Effects of natural polyphenol-rich pomegranate juice supplementation on plasma ion and lipid profiles following resistance exercise: a placebo-controlled trial

**DOI:** 10.1186/s12986-020-00451-1

**Published:** 2020-04-16

**Authors:** Achraf Ammar, Khaled Trabelsi, Stephen J. Bailey, Mouna Turki, Nicola Luigi Bragazzi, Omar Boukhris, Kais El Abed, Mohamed Bouaziz, Fatma Ayadi, Tarak Driss, Nizar Souissi, Hamdi Chtourou, Anita Hökelmann

**Affiliations:** 1 Institute of Sport Sciences, Otto-von-Guericke University, Magdeburg, 39104 Germany; 2 Unit of Research Molecular Bases of Human Diseases, 12ES17, Faculty of Medicine of Sfax, University of Sfax, Sfax, 3000 Tunisia; 3 High Institute of Sport and Physical Education, University of Sfax, Sfax, 3000 Tunisia; 4 Research Laboratory: Education, Motricité, Sport et Santé, EM2S, LR19JS01, High Institute of Sport and Physical Education of Sfax, University of Sfax, Sfax, Tunisia; 5 School of Sport, Exercise and Health Sciences, Loughborough University, Loughborough, LE11 3TU UK; 6 Laboratory of Biochemistry, CHU Habib Bourguiba, Sfax University, 3000 Sfax, Tunisia; 7 Department of Health Sciences (DISSAL), Postgraduate School of Public Health, University of Genoa, Genoa, 16132 Italy; 8 Department of Mathematics and Statistics, Laboratory for Industrial and Applied Mathematics (LIAM), York University, Toronto, ON M3J 1P3 Canada; 9 Activité Physique, Sport et Santé, UR18JS01, Observatoire National du Sport, Tunis, 1003 Tunisia; 10 High Institute of Biotechnology, Sfax University, Sfax, 3000 Tunisia; 11 Interdisciplinary Laboratory in Neurosciences, Physiology and Psychology: Physical Activity, Health and Learning (LINP2-2APS), UFR STAPS, UPL, Paris Nanterre University, Nanterre, 92000 France

**Keywords:** Supplementation, Pomegranate juice, Physical exercise, Weightlifting

## Abstract

**Background:**

Pomegranate juice (POMj) contains abundant soluble polyphenolic antioxidant compounds and is recommended for its cardioprotective/atheroprotective properties. However, very few studies have investigated the efficacy of POMj supplementation to alter physiological responses during intensive physical exercise. This placebo-controlled study aimed to examine whether supplementation with natural polyphenol-rich-POMj could influence the ionic or lipid responses to an intensive resistance training session in elite athletes.

**Methods:**

Nine elite weightlifters (21 ± 1 years) performed two Olympic-weightlifting sessions after ingesting placebo and POMj supplements. Venous blood samples were collected at rest and 3 min after each session for assessment of plasma sodium ([Na^+^]), potassium ([K^+^]), chloride ([Cl^−^]), calcium ([Ca^2+^]), triglyceride ([TG]) and high-density lipoprotein ([HDL-C]), low-density lipoprotein ([HDL-C]) and total ([TC]) cholesterol concentrations.

**Results:**

Plasma [K^+^] and [TG] were lowered post-exercise compared to resting values in the PLA condition (*p* = 0.03 for K^+^ and *p* = 0.02 for TG) with no pre-to-post exercise differences in the other plasma ion and lipid markers (*p* > 0.05). Compared to rest, plasma [Na^+^] and [Cl^−^] were increased (*p* = 0.04, %change = 4.10% for Na^+^ and p = 0.02, %change = 4.44% for Cl^−^), but there were no differences in the other plasma ion or lipid markers post-exercise after POMj supplementation (*p* > 0.05). Post-exercise plasma [Na^+^], [Cl^−^], and [HDL-C] were greater following POMj supplementation compared to PLA (*p* = 0.01 for Cl- and HDL-C, *p* = 0.02 for Na+, and *p* = 0.04 for TC), with no between-supplement post-exercise differences in the other ion and lipid markers (*p* > 0.05).

**Conclusion:**

In conclusion, supplementation with POMj has the potential to attenuate the acute imbalance of plasma [K^+^] and to improve blood lipid responses (i.e., HDL-C) following resistance exercises in elite weightlifters. However, further large research in both athletic and non-athletic populations is needed to corroborate these preliminary observations and to elucidate the potential underlying mechanisms and translational potential of our novel observations.

**Trial registration:**

**Name of the registry:**ClinicalTrials.gov PRS

**The registration number:**NCT02697903.

**Date of Registry**: 03/03/2016 ‘Retrospectively registered’.

**The registration title:** Pomegranate Improve Biological Recovery Kinetics in Elite Weightlifter.

**Graphical abstract:**

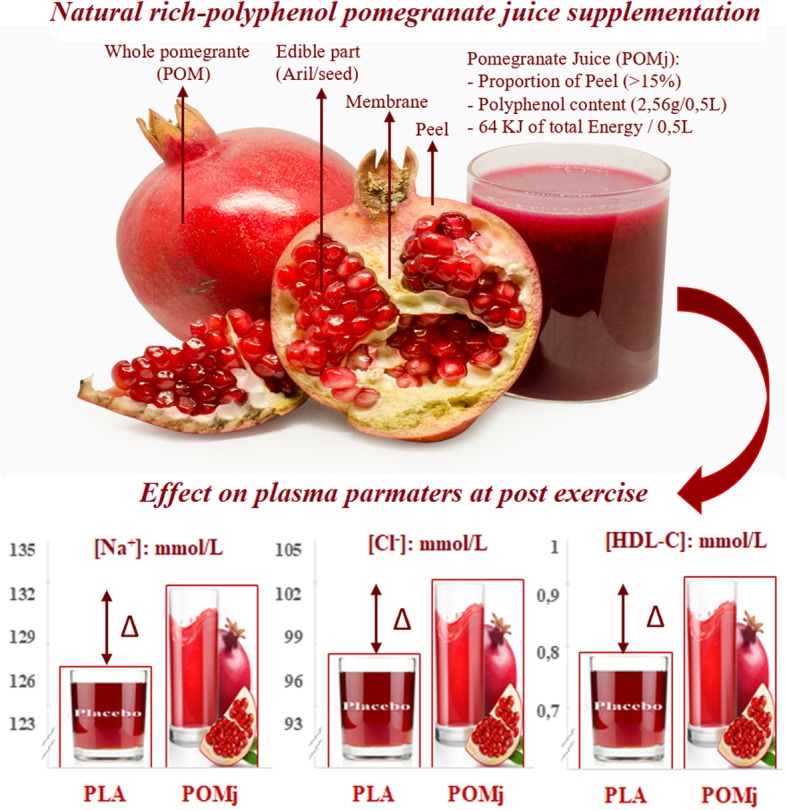

## Background

Ions are positively (cations) or negatively (anions) charged molecules that are ubiquitous in the human body [1–3]. Proper electrolyte balance in the body is essential for normal functioning of cells, while alterations in electrolyte balance can impair cardiovascular and thermoregulatory functions [4]. With regard to exercise performance and post exercise recovery, sodium (Na^+^), potassium (K^+^), chloride (Cl^−^), calcium (Ca^2+^) and magnesium (Mg^2+^) are the most important electrolytes [1, 4]. In the extracellular fluid, Na^+^ is the primary cation and helps regulate acid base balance, nerve conduction, blood pressure, and muscle function [1, 5]. Cl^−^ is the primary anion and works in tandem with sodium to regulate nerve impulse conduction and body water balance [6]. In intracellular fluid, K^+^ is the primary cation and helps maintain electrical activity in nerves, skeletal muscles, and the heart and also aids carbohydrate metabolism by enhancing glucose transport and glycogen storage [7, 8]. Ca^2+^ and Mg^2+^ are cationic electrolytes that play important roles in the regulation of muscle contraction and enzymatic reactions [9–11]. In order to maintain skeletal muscle contractility and force production during exercise, the concentrations of several ions (i.e., Na^+^, K^+^, Cl^−^, Ca^2+^) change simultaneously in interstitial, transverse tubular and intracellular compartments [1, 12, 13].

Although, these acute ionic adjustments are obligatory for the contraction of skeletal muscle, the development of ionic imbalances during repeated skeletal muscle contractions is implicated in the process of skeletal muscle fatigue [1, 5, 9]. Indeed, a diminished trans-sarcolemmal K^+^ gradient coupled with lowered Na^+^, Ca^2+^ and Cl^−^ gradients can impair force production and cause fatigue [13]. Maintenance of ion homeostasis is, therefore, integral for optimizing skeletal muscle function, exercise performance and post-exercise recovery.

Previous studies investigating plasma ion responses following prolonged, high-intensity exercise (e.g., soccer) or exercise in the heat have reported slight to significant hyper- or hypokaliemia (high- or low [K^+^]) and slight to significant hyper- or hyponatremia (high- or low or [Na^+^]) [2, 14–17]. Discrepancies between findings might be linked to the configuration of the exercise protocol administered (e.g., intensity and duration) [14, 15, 18], with high intensity (e.g., sprint, cycling) [2, 7] and/or more prolonged exercise (e.g., 100 km run, marathon, prolonged submaximal cycling) [15, 16] resulting in greater blood ion imbalances (e.g., Hyper- kaliemia or natremia). Indeed, in response to short duration moderate exercise (45 min), Emenike et al. [17] showed only a slight reduction in serum [K+] and [Na+], while short maximal isokinetic cycle ergometer exercise [2] and prolonged submaximal-cycling [15] or moderate-running [16] exercise were associated with significant increases in plasma [K+] and [Na+]. These pronounced increases in plasma [K+] and [Na+] following prolonged and/or intensive exercises have been suggested to be due to the higher loss of fluids (via sweat) during prolonged exercise and to the inactivation of some Na^+^-K^+^-ATPase pumps, which play a crucial role in stabilizing Na + and K+ concentration gradients and membrane excitability) during intense contractions [19]. However, although resistance exercise (e.g., weightlifting) is clearly classified as high-intensity physical exercise [20–26], there is a dearth of available studies addressing potential ionic imbalances during such exercise, and the existing evidence is conflicting [16, 17].

Physical exercise is also known to bring about changes in the blood lipid profile [27]. In term of improving athlete’s cardiovascular health, an increase in high-density lipoprotein cholesterol (HDL-C), and reductions in low-density lipoprotein cholesterol (LDL-C), triglycerides (TG) and total cholesterol (TC) are classified as favorable post-exercise changes [28]. Nevertheless, such favorable alterations in blood lipid profile after a single session of exercise is not always observed [29, 30]. The available studies regarding the acute effect of physical exercise on plasma lipid profile have especially focused on prolonged aerobic based exercise and showed contradictory findings (i.e., decrease in LDL and increase in HDL [31, 32] vs. no change in both biomarkers [28, 33]). Only two studies [14, 34] have been conducted to evaluate the influence of acute resistance exercise on post-exercise blood lipid profile and reported equivocal findings with a favorable increase of HDL-C registered only in the study of Hill et al. [34]. Therefore, given that lipid profile as well as plasma electrolytes responses are not yet established during intensive resistance exercises, it seems appropriate to investigate whether intensive weightlifting training-session can impair plasmatic electrolyte and lipids balance in elite athletes.

There is emerging evidence to suggest that dietary supplementation with food products rich in polyphenols might improve cardiometabolic risk factors such as hypertension and the lipid profile [35–38]. For example, pomegranate juice (POMj) contains abundant soluble polyphenolic antioxidant compounds and is recommended as a dietary intervention to confer cardioprotective / atheroprotective effects, including lowering TC and inhibiting LDL oxidation [37, 38]. Indeed, the anthocyanin- and ellagitannin-active constituents of POMj can protect LDL against cell-mediated oxidation via the scavenging of reactive oxygen and nitrogen species or through their accumulation in arterial macrophages [15, 39]. Moreover, since Na^+^-K^+^-ATPase pump [15] and Ca^2+^-ATPase pump [40] activity can be compromised by oxidative stress, POMj supplementation might blunt the ionic imbalances that develop during high-intensity exercise.

The purpose of the current study was to assess the effect of POMj supplementation on blood ions and lipid profiles following a resistance exercise training session. It was hypothesized that high-intensity resistance exercise would elicit blood ionic imbalances and positively modulate the blood lipid profile, and that POMj supplementation would abate blood ionic imbalances and promote further improvements in the blood lipid profile compared to a placebo condition.

## Methods

The authors confirm that all ongoing and related trials for this intervention are registered.

### Participants

The sample size was calculated a priori, using procedures described by Beck [41] and the software G*Power [42]. Values for α were set at 0.05 and for power at 0.90. Based on the results of Beyer et al. [43] and Ammar et al. [25], effect sizes were estimated to be 0.53 (medium effect). To reach the desired power, data from at least eight participants were deemed to be sufficient to minimize the risk of incurring a type 2 statistical error.

Nine healthy elite male weightlifters participating in a regional or national team and competing at an international standard [age: 21 ± 1 years, weight: 80 ± 10 kg, height: 1.75 ± 0.08 m, BMI: 23.4 ± 1.17, one-repetition maximum (1RM) Clean & Jerk: 150 ± 7 kg, 1RM Snatch: 120 ± 6 kg (mean ± SD)] volunteered to participate in this study. Potential participants were initially screened through telephone interviews based on the following inclusion criteria: i) 18–26 years of age, ii) body mass index (BMI) less than 25 kg/m^2^, iii) they trained at least six sessions per week with at least 5 years’ experience of Olympic weightlifting and iv) they did not have any injury or any other health problems. Exclusion criteria included: i) diagnosis of any chronic metabolic disease such as type 2 diabetes or cardiovascular disease, ii) diagnosis of an auto-immune disease such as rheumatoid arthritis, lupus, or type 1 diabetes, liver disease and iii) the intake of any medications (e.g., antioxidant or anti-inflammatory drugs) or dietary supplements (e.g., creatine, foods rich in antioxidants or polyphenols such as blueberries, coffee, tea, grapes, cherries, curcuma, red wine and dark chocolate) during the experimental period and for at least one month before the commencement of the study. After receiving a thorough explanation of the possible risks and discomforts associated with the experimental procedures, each participant provided written informed consent to take part in the experiment. The study was conducted according to the Declaration of Helsinki and the study’s protocols and procedures were fully approved by the local ethics committee of the CHU Habib Bourguiba, university of Sfax, Tunisia, before the commencement of the assessments (identification code: 8/16). Additionally, all ongoing and related trials for this intervention were registered with Clinical Trials.gov (identification code: NCT02697903).

### Experimental design

**Fig. 1:**
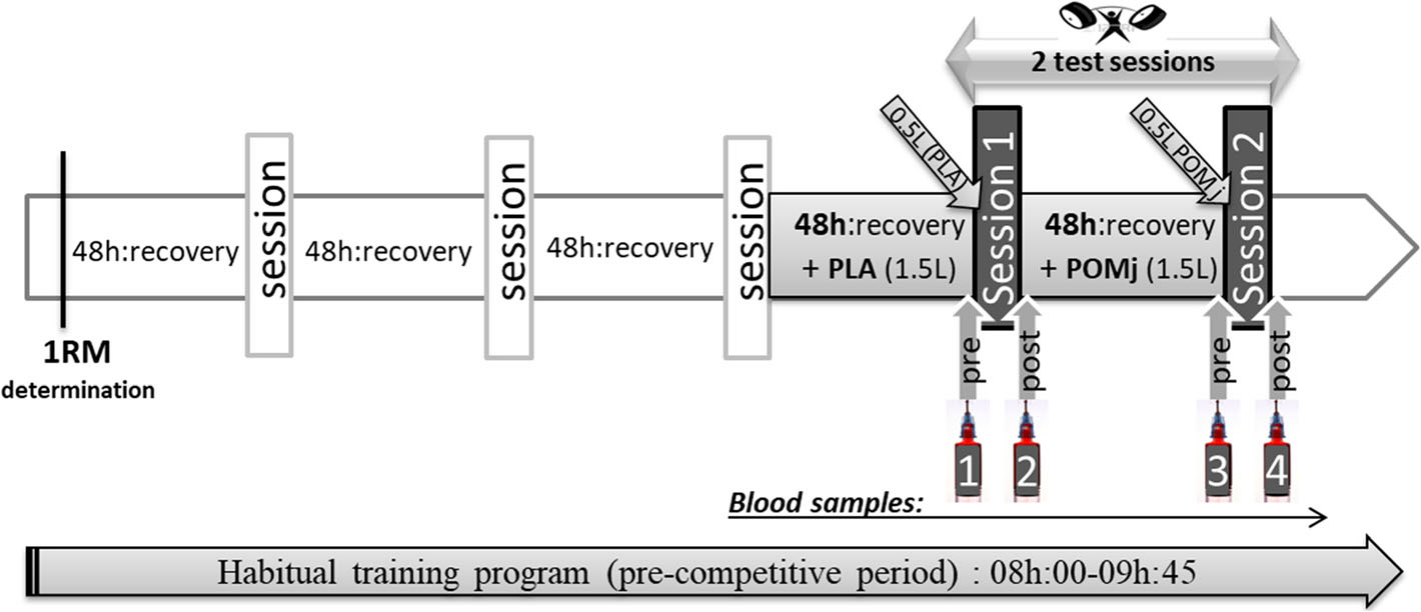
Experimental design

A non-randomized placebo-controlled design was adopted for this study (Fig. 1). Neither staff nor participants were informed about the names of the two drinks (POMj and placebo, PLA), and blinding was strictly maintained by emphasizing to both staff and participants that both drinks were health promoting and advocated as potentially performance enhancing by certain sports medicine experts.

One week before the start of the experimental period, the heaviest weight lifted in a single repetition (1-RM) was assessed for each participant in each Olympic movement (Fig. 1). 1-RM was determined in three trials interspersed by 5 min recovery after an ascending warm-up from 40 to 80% of the athlete’s estimated maximum [26]. Thereafter, participants performed, as part of their habitual training-program (i.e., during the pre-competitive period) to avoid any repeated bout effect, two training sessions (Fig. 1) from 08:00 to 09:45 following the consumption of PLA and POMj with a 48 h washout between conditions [23, 25]. Before and 3 min after each training session, blood samples were collected (Fig. 1). Each resistance training session comprised three Olympic Weightlifting exercises: snatch, clean and jerk, and squat (performed in the same order for all participants and for both treatments) with 5 sets for each exercise and a total session duration of 1 h 46 min. Specifically, participants completed 2 sets of 3 repetitions at 85% of 1-RM and 3 sets of 2 repetitions at 90% of 1-RM. A passive recovery period of 5 and 8 min was administered between sets and the different Olympic-Weightlifting exercises, respectively.

Participants consumed 250 mL of the PLA or POMj supplements three times daily (i.e., 07 h:00, 15 h:00 and 23 h:00) over the 48 h that preceded the two training sessions. To ensure that each subject consumed the correct dose at the correct time point, subjects were contacted through a mobile telephone. Moreover, participants consumed an additional opaque and unmarked 500 mL can of PLA or POMj 60 min before commencing the training sessions to facilitate beneficial physiological effects [24]. Subjects were supervised by staff to ensure that they drank the entire quantity of fluid, and no exchange of bottles was allowed. Participants were instructed to drink the fluid quickly (within 1 ± 0.5 min) 60 min before their test session and not to discuss or compare tastes or to make any assumptions about what they had ingested. Consumption of POMj 60 min prior to training was chosen as this has been reported to be optimal for a complete polyphenol absorption, thereby favoring the attainment of peak polyphenol concentrations [24]. Beverages were prepared by an agri-food engineer. The natural POMj was prepared from a fresh pomegranate fruit 48 h before the beginning of the experimentation and was stored at − 4 °C. No additional chemical products were added to the natural POMj. PLA juice consisted of a pomegranate-flavored drink containing mineral water, natural identical flavor (pomegranate), stabilizers (Arabic-gum) with caloric sweeteners (i.e., added sugar) added to match the POMj energy content and avoid any confounding effect of different caloric content of the beverages [44]. The two drinks were similar in volume, texture, and appearance. One can of POMj (i.e., 500 mL) contained 2.56 g of total polyphenols, 1.08 g of orthodiphenols, 292.6 mg of flavonoids and 46.75 mg of flavonols, 64 g of total carbohydrates (i.e., 56 g of sugar), and 1046 kJ of energy. The PLA drink (i.e., 500 mL) did not contain antioxidants, vitamins or polyphenols, but comprised 60 g total carbohydrates (i.e., 54 g of added sugar), and 983 kJ of energy (Table 1). More details regarding the POMj processing [22, 45, 46], phenolic extracts [47] and the determination of the total phenolic content [48, 49] can be found in supplementary materiel 1.

**Table 1:** Nutrition Facts of Pomegranate and Placebo Juices

Variables	Contents / 500 ml	
	POMj	PLA
Total polyphenols (g)	2,56	0
Orthodiphenols (g)	1,08	0
Flavonoids (mg)	292,6	0
Flavonols (mg)	46,75	0
Calories	250	235
Calories from Fat	0	0
Total Fat (g)	0	0
Saturated Fat (g)	0	0
Trans Fat (g)	0	0
Cholestreol (mg)	0	0
Sodium (mg)	15	12
Potassium (mg)	1270	4
Calcium (mg)	48	46
Total Carbohydrate (g)	64	60
Dietery Fiber (g)	0	0
Sugars (g)	56	54
Protein (g)	< 1	0
Vitamin A (%)	0	0
Vitamin C (%)	0	0
Iron (%)	0	0

Before test sessions, participants underwent an overnight fast and were only permitted to drink one glass of water (15–20 cL, depending on body mass: 0.22cL / Kg of body mass) in the morning to avoid the potential confounding influence of postprandial thermogenesis [50, 51].

Given that randomly assigning the supplements would have resulted in some participants consuming the POMj supplement before the PLA supplement, and since the beneficial effects of POMj could persist for up to three weeks after consumption [52], the authors elected to avoid any potential confounding effect of POMj supplementation altering blood biochemistry responses during the PLA condition by ensuring all participants completed the PLA condition first as recently suggested by Ammar et al. [24]. As the participants were elite weightlifters who were well trained and familiar with the exercises, and as the protocol (i.e., including both PLA and POMj conditions) was completed by all participants during the regular training program in the middle of the precompetitive period, the authors assume that an order effect is less likely to occurred. To avoid any time of day effects [20–22], all sessions were arranged in the morning hours.

### Dietary records

To assess the adequacy of nutrient intake, a consecutive dietary record over the experimental period was completed. All participants received a detailed verbal explanation and written instructions on how to record their diet over the study period. Participants were asked to continue with their usual dietary habits during the period of dietary recording, but instructed to avoid antioxidant or anti-inflammatory drugs, and nutrients, foods or beverages rich in antioxidants, polyphenols or vitamins (e.g., blueberries, coffee, tea, grapes, cherries, curcuma, red wine and dark chocolate etc.). Indeed, a detailed list of such nutrients was given to all participants and they were asked to avoid consumption of such products from one month before experimentation and during the experimentation with a daily reminder via phone call during the experimental period. Additionally, participants were asked to be as accurate as possible in recording the amounts and types of food and fluid consumed. A list of common household measures, such as cups and tablespoons, and specific information about the quantity in each measurement (grams, etc.) were given to each participant. Each individual’s dietary composition was calculated using the Bilnut 4 software package (SCDA Nutrisoft, Cerelles, France) and the food composition tables published by the Tunisian National Institute of Statistics in 1978.

### Blood sampling and analysis

Blood samples (7 mL) were collected from a forearm vein at rest and 3 min following each training session in the PLA and POMj conditions. Samples were placed in an ice bath and immediately centrifuged at 3000 rpm 1008 RCF and 4 °C for 15 min. Aliquots of the resulting plasma have been analyzed in the same day and in the same assay run to eliminate inter-assay-variance. All assays were performed in duplicate in the same laboratory with simultaneous use of a Multichem control serum. Levels of plasma ions and lipid profile parameters were determined spectrophotometrically (Architect Ci-4100-ABBOTT, Abbott Deutschland, Wiesbaden, Germany) using the indirect potentiometric method for [Na^+^], [K^+^] and [Cl^−^], the single stable reagent (Arsenazo III) method for [Ca^2+^], the enzymatic hydrolysis method by cholesterol esterase for [TC], the enzymatic hydrolysis and oxidation method by glycerol phosphate oxidase for [TG], and the enzymatic method by selective detergent for [HDL-C]. Plasma LDL-C contents have been estimated by Friedewald equation: [LDL-C] = [TC] - ([HDL-C] + [TG/2,2]). Additionally, to account for any change in plasma volume shifts post exercise, hematological parameters [i.e., Red Blood Cells (RBC), Hemoglobin (HB), Hematocrit (HCT)] were assessed within 3 h in a multichannel automated blood cell analyzer (Beckman Coulter Gen system-2, Coulter T540, California, United States). As [HB] and [HCT] are highly sensitive to posture changes, all blood samples were collected in the sitting position after 5 min resting time for baseline (pre-exercise) samples and 3 min resting time for post exercises samples.

### Statistical analyses

All statistical analyses were performed using STATISTICA 10.0 Software (StatSoft, Maisons-Alfort, France). Normality of the data distribution was confirmed using the Kolmogorov-Smirnov test (*p* > 0.2 for Na^+^, K^+^, Cl^−^, Ca2^+^, TC, and TG, and *p* = 0.09 for HDL-C and LDL-C). To analyze the effect of POMj supplementation on the plasma ions and lipid profile responses during training sessions (pre-post values), a two-way [supplement (PLA and POMj) × time (pre and 3 min post training session)] ANOVA with repeated measures was employed. When significant effects were observed, Tukey’s honest-significance-difference (HSD) post-hoc tests were conducted. Effect sizes were calculated as partial eta-squared (η_p_^2^) for the ANOVA analysis to interpret the magnitude of the change score using the following criteria: < 0.2 = trivial, 0.2–0.6 = small, 0.6–1.2 = moderate, 1.2–2.0 = large, 2.0–4.0 = very large, and > 4.0 = extremely large [53]. Statistical significance was set at *P* < 0.05 and data are presented as mean ± SD unless otherwise stated.

## Results

### Dietary records

Estimated nutrient intakes were referred to reference dietary intakes for physically active people and there were no differences in total calorie, macronutrient, and micronutrient intakes between the PLA and POMj conditions (Table 2).

**Table 2:** Dietary record of the subjects (mean ± SD)

Variables	Mean ± SD	
	PLA testing days	POMj testing days
Kilocalorie	3269 ± 485	3254 ± 510
CHO (%)	53.63 ± 5.6	52.76 ± 5.2
Protein (%)	11.83 ± 1.1	12.37 ± 1.3
Fat (%)	27.09 ± 4.0	27.42 ± 4.2
Cholesterol (mg·day− 1)	326.1 ± 97	314.8 ± 65
Vit C (mg·day− 1)	45.33 ± 11	44.97 ± 13
Vit E (mg·day− 1)	4.10 ± 0.8	4.07 ± 0.6
Vit A (ER)	1300 ± 252	1280 ± 189
Folate (μg·day− 1)	335.6 ± 53	329.8 ± 49
Vit B12 (μg·day− 1)	7.1 ± 2.0	6.9 ± 1.9

### Hematological parameters cell count

Compared with the baseline values, there was no significant changes in the hematological parameters in response to the resistance exercises in both PLA and POMj conditions (Table 3). Additionally, there was no significant differences between supplementation conditions at any of the time-points. Therefore, any observed changes in the blood parameters assessed (i.e., circulating ions and lipid profile) in response to the resistance exercises or POMj supplementation cannot be attributed to changes in hemoconcentration [54].

**Table 3:** Hematological parameters before, immediately (3 min) and 48 h after the PLA and POMj resistance training sessions

Parameters	PLA		POMj	
	Basal	3 min	Basal	3 min
***RBC***	5.60	5.68	5.43	5.51
***(10***^***6***^***/ μl)***	±0.21	±0.18	±0.14	±0.20
***Hemoglobin***	15.68	16.30	15.96	16.06
***(g/dl)***	±1.33	±1.95	±1.36	±1.49
***Hematocrit***	48.53	48.94	47.71	48.19
***(%)***	±2.02	±2.57	±2.10	±2.36

Placebo (*PLA*), Pomegranate juice (*POMj*) and Red Blood Cells (*RBC*)

### Acute effect of POMj on plasma ions concentrations following a resistance training session

**Fig. 2:**
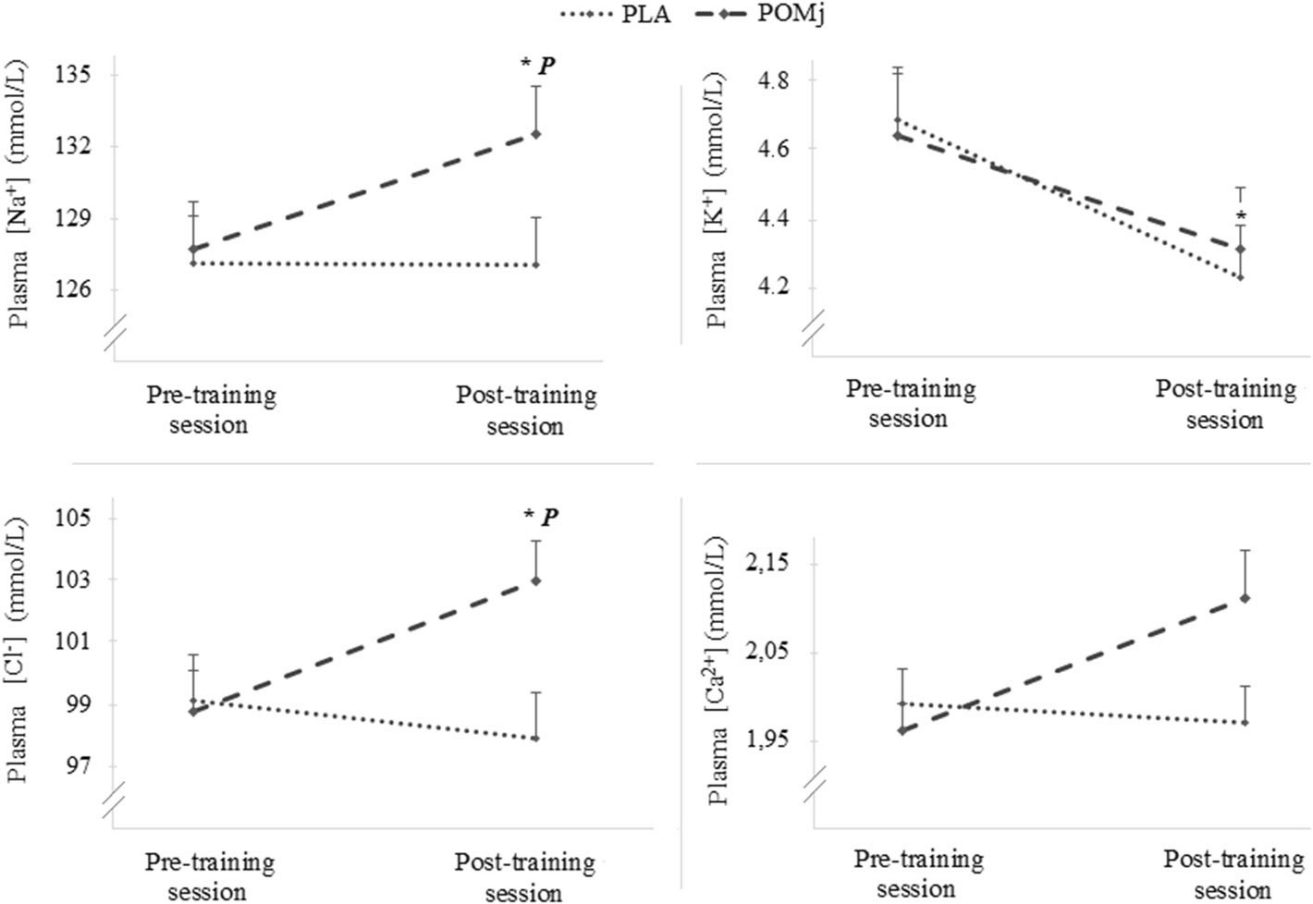
Acute plasma ions in response to a resistance training session following POMj and PLA supplementation. *: Significant differences between pre- and 3 min post- training session with *p* < 0.05. P: Significant difference between PLA and POMj condition with p < 0.05. Placebo (PLA), Pomegranate juice (POMj), Sodium (Na+), Potassium (K+), Chloride (Cl) and Calcium (Ca2+)

Mean values for the blood ions (i.e., Na^+^, K^+^, Cl^−^ and Ca^2+^) before and 3 min after the resistance training sessions (i.e., in the PLA and POMj conditions) are presented in Fig. 2. Statistical analysis showed a significant main effect of supplementation on [Na^+^] (F_(1,8)_ = 5.57, *p* = 0.04, η_p_^2^ = 0.41) and [Cl^−^] (F_(1,8)_ = 6.37, *p* = 0.03, η_p_^2^ = 0.44) with a significant increase pre-post training session only observed during the POMj condition (i.e., *p* = 0.04, % change = 4.10 ± 2.37% for [Na^+^] and *p* = 0.02, % change = 4.44 ± 2.03% for [Cl^−^]), resulting in higher post-session values of [Na^+^] and [Cl^−^] during the POMj condition compared to the PLA condition (p = 0.02 and *p* = 0.01, respectively).

A significant main effect of time was observed for [K^+^] (F_(1,8)_ = 5.65, p = 0.04 η_p_^2^ = 0.41) with plasma [K^+^] significantly decreasing from pre-to-post- training session only during the PLA condition (*p* = 0.03, %, change = − 8.77 ± 4.19%). However, there was no significant difference in plasma [K^+^] between conditions 3 min post training session (*p* > 0.05). Similarly, no significant effect of supplementation condition or time was observed for [Ca^2+^] during the resistance training session (p > 0.05).

### Acute effect of POMj on plasma lipid concentrations following a resistance training session

**Fig. 3:**
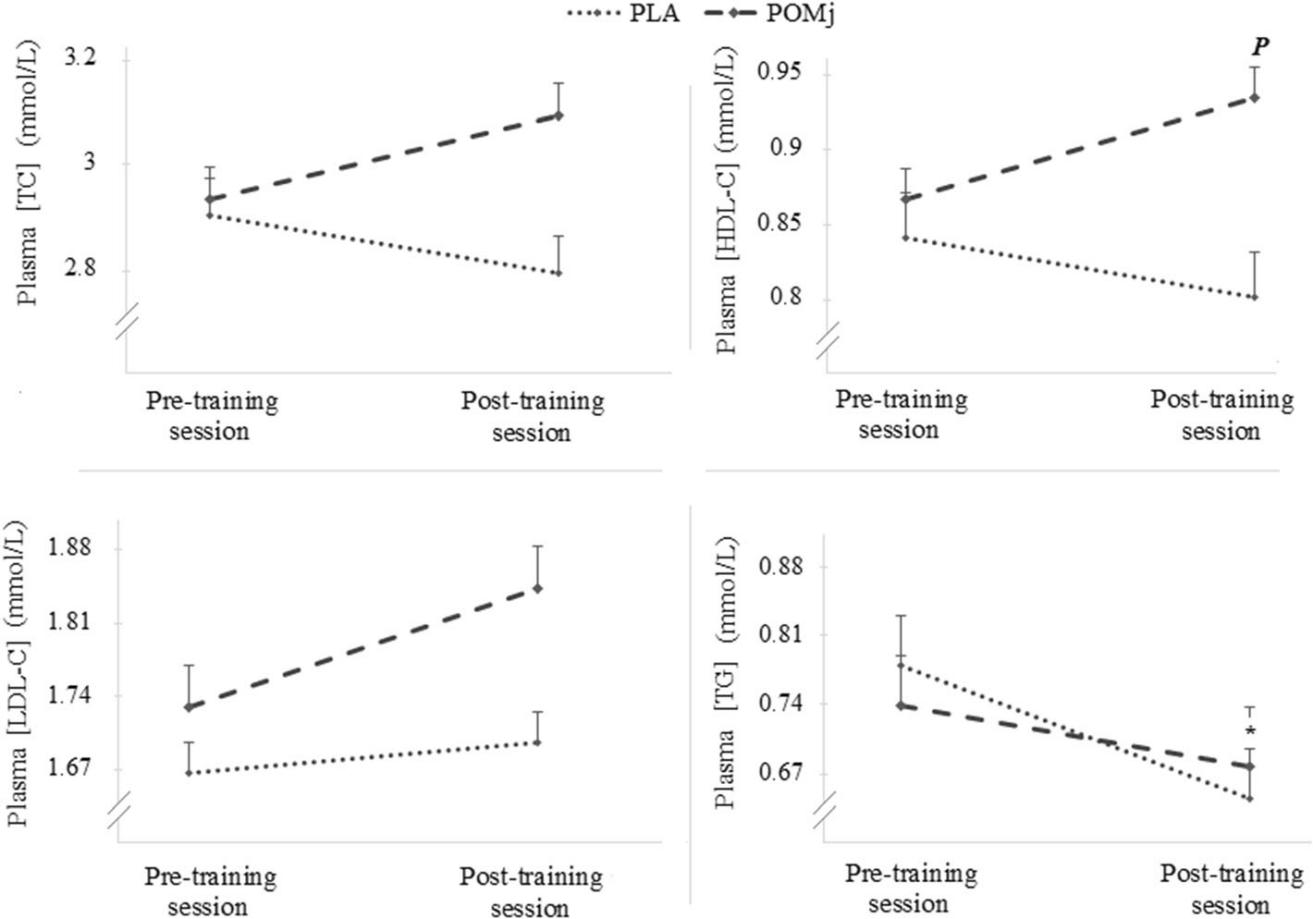
Acute blood lipid profile responses to a resistance training session following POMj and PLA supplementation. *: Significant differences between pre- and 3 min post- training session with p < 0.05. ***P***: Significant difference between PLA and POMj condition with p < 0.05. Placebo (PLA), Pomegranate juice (POMj), Total Cholesterol (TC), High-density Lipoprotein cholesterol (HDL-C), Low-density Lipoprotein cholesterol (LDL-C), Triglycerides (TG)

Fig 3 shows the plasma lipid profile (i.e., TC, HDL-C, LDL-C and TG) at pre- and post-training session during both PLA and POMj conditions. A significant main effect of supplementation condition was found for HDL-C (F_(1,8)_ = 6.85, p = 0.03, η_p_^2^ = 0.46) with higher values 3 min post-training session in the POMj compared to the PLA conditions (*p* = 0.01). A significant pre-post training session decrease was observed for TG during the PLA condition with *p* = 0.02 and % change = − 12.79 ± 6.59%. There was no significant main effect of supplementation condition on TC, TG or LDL-C (p > 0.05).

## Discussion

The aim of the present study was to investigate the effect of POMj supplementation on the responses in blood ions and lipids following an acute high-intensity resistance training session. In the PLA condition, plasma [K^+^] and [TG] were lower following the training session compared to resting baseline values. However, there were no pre-to-post-exercise differences in plasma [K^+^] and [TG] following POMj consumption, and post-exercise plasma [Na^+^], [Cl^−^], and [HDL-C] were greater following POMj supplementation compared to PLA supplementation. However, only limited ionic and lipid imbalance was evoked by resistance exercise in the current study as evidenced by alterations in some (K^+^ and TG), but not all, ion and lipid markers. Since POMj supplementation only modulate post-exercise plasma [Na^+^], [Cl^−^] and [HDL-C] compared to PLA, the potential for POMj supplementation to improve resistance exercise performance and cardiometabolic health in trained athletes appears to be limited.

There is a strong body of evidence indicating that ionic imbalances during and following physical exercise is linked to the type of exercise administered and the environmental conditions [3, 12]. Following the high-intensity resistance exercise completed in normothermic conditions in the present study, plasma [K^+^] was lower compared to baseline in the PLA condition. However, plasma [Na^+^], [Ca2^+^] and [Cl^−^] were not altered after the high-intensity resistance exercise protocol in the PLA condition. The findings of the present study are in line with those of Emenike et al. [17] with regard [K^+^], but conflicts with the observations from this study regarding [Na^+^] and [Cl^−^] responses. Indeed, the short duration exercise (45 min) investigated by Emenike et al. [17] was associated with a slight reduction in serum [K^+^], [Na^+^] and [Cl^−^]. Similarly, the present results conflict with numerous previous studies reporting increased plasma ions during 30 s maximal isokinetic cycle ergometer exercise (i.e., [K^+^], [Na^+^] and [Cl^−^], [2]), 90 s exhaustive intense knee-extensor exercise (i.e., [K^+^], [55]) and prolonged submaximal-cycling (i.e., [K^+^], [Na^+^] and [Ca^2+^], [15]) or moderate-running (i.e., [K^+^] and [Na^+^], [16]) exercise.

Previous reports have suggested that the inter-study discrepancies in plasma ion responses to physical exercise is linked to the configuration of the exercise protocol administered (e.g., intensity and duration) [14, 15, 18], with high intensity [15] and/or more prolonged exercise [16, 17] resulting in greater blood ion imbalances. However, the significant decrease of plasma [K^+^] and the absence of plasma [Na^+^], [Ca^2+^] or [Cl^−^] alterations following the intensive long duration (1 h 46 min) resistance session administered in the current study suggests that other factors are involved in mediating the ionic response to exercise. For example, the participant’s training status could influence the effect of high-intensity exercise on plasma ionic responses. Indeed, increased plasma ion concentrations have been observed in studies investigating healthy habitually active [2, 15, 55] or amateur subjects [16] while the attenuated ionic responses in the present study and in the study of Emenike et al. [17] have been observed in more highly trained participants. In this context, it has previously reported that well trained subjects undergo lower physiological (i.e., ionic imbalances, muscle damage, oxidative stress, inflammation etc) perturbations in responses to physical exercise [2, 20–26] due to the chronic adaptation of numerous physiological processes. Indeed, after 7 weeks of sprint training, McKenna et al. [2] showed that maximal exercise was accompanied by lower femoral arterial and venous plasma [K^+^] and [Na^+^] across all measurement times points (i.e., 0 to 10 min post exercises) compared to pre-training. These blunted changes in plasma [K^+^] and [Na^+^] is likely the result of a greater activation of the muscle Na^+^/K^+^-pump.

With regard to skeletal muscle function, it was previously reported that acute ionic adjustments are obligatory to maintain skeletal muscle contractility and force production [5, 12, 56]. In this context, it was shown that the Na^+^/K^+^ pump activity increases more than 20-fold in order to maintain Na^+^ and K^+^ concentration gradients during exercise [12] with exercise-induced lactic acidosis increasing extra-cellular Cl^−^ to help maintain skeletal muscle excitability [57]. However, during repeated skeletal muscle contractions (e.g., prolonged exercise) and/or high intensity contraction, these adjustments can lead to the development of ionic imbalances which is implicated in the process of skeletal muscle fatigue and may adversely affect exercise performance [1, 5, 9]. In the present study, there were no alterations in the majority of the ions assessed (i.e., [Na+], [Cl-] and [Ca2+]) between pre- and post-exercise in PLA condition during a single bout of high-intensity resistance exercise performed by elite athletes. This limited ionic imbalance is likely due to the high training status of the participants ([2, 20–26]) and suggests a limited role of ionic adjustment on exercise performance for this specific population in this context.

The administration of POMj in the present study blunted the decline in plasma [K^+^] and increased post-exercise plasma [Na^+^] and [Cl^−^] compared to the PLA condition, but did not lead to hypernatremia ([Na+] > 145 mmol/l [58]) abnormality. Although, the potential functional consequences of this effect are unclear, the higher plasma [Cl^−^] and the stabilization of [K^+^] response using POMj compared to PLA may suggest promising potential of POM supplementation in modulating muscle excitability, physical performance and post exercise fatigue. Indeed, increased sarcolemma Cl^−^ permeability and extra-cellular Cl^−^ levels has previously been shown to help maintain skeletal muscle excitability [57]. Likewise, maintained [K^+^] has been shown to (i) play a key role in maintaining muscle contraction during exercise by helping transport glucose into the muscle cells [5], (ii) interact with both Na^+^ and Cl^−^ to control fluid and ionic balances, and (iii) assist in the conduction of nerve impulse and muscle contractility [8, 12, 59].

The mechanism underlying the effects of POMj supplementation on plasma ions during exercise remains to be elucidated [24]. However, it is acknowledged that the present attenuation in the decline in K^+^ concentration from pre- to post- exercise using POMj compared to PLA could be explained by the higher K^+^ content in the POMj used (1.27 g/500 ml) compared to PLA (only trace). Previous studies in health and disease have linked the observed ionic changes using POM consumption to the protective role of POM-polyphenols against reactive oxygen species (ROS)-induced inhibition of Na^+^/K^+^ pump activity in various tissues including brain, kidney and myocardium [60–62]. However, this potential beneficial effect of consuming POM-polyphenols on Na^+^/K^+^ pump activity has yet to be confirmed in skeletal muscle tissue following high-intensity exercise.

Concerning the lipid profile, in response to the investigated high-intensity resistance exercise, plasma [TG] declined post-exercise, with no change in plasma [TC], [HDL-C] or [LDL-C] compared to pre-exercise during the PLA condition. Previous studies focusing on the acute responses of blood lipid profile following strength exercises reported divergent findings with Lahiji et al. [28] showing no significant change in lipid profile immediately after one resistance exercise session, while a significant increase of HDL-C was immediately shown following a session with a similar training-volume but a higher intensity [34]. Discrepancies between these findings suggest that the lipid profile response to a single session of resistance exercise might be more influenced by exercise intensity [34]. Indeed, it has been recently suggested that intensive exercise may alter lipid profile by increasing expression of ATP-binding cassette transporter A-1 (ABCA1) in macrophages which has a strong effect on promoting the reverse cholesterol transport (RCT) pathway, plasma HDL-C formation, and protection against atherosclerosis [63]. The significant increase of [HDL-C] previously shown following intensive resistance exercises performed by a non-athlete population [28] and the attenuation of [TG] content among elite athlete population in the present study are in line with this suggestion.

Regarding the effect of POMj on lipid profile response, the significantly higher content of HDL-C at post training session following POMj supplementation compared to PLA confirm the potential improvement in cardiometabolic disease risk factors following POMj supplementation, as previously highlighted by Al-Dujaili et al. [64, 65] in healthy volunteers, and most importantly in vascular disease therapy [36, 38]. Indeed, in patients with both diabetes and hyperlipidemia, the administration of 40 g concentrated POMj for 8 weeks was shown to decrease [TC], [LDL-C], LDL-C/HDL-C ratio and TC/HDL-C ratio [66]. Similarly, the administration of 200 ml/day POMj for 6 weeks, in patients with type 2 diabetes, decreased [TC] and [LDL-C] [38]. In relation to physical exercise, the present study is the first to test the potential of POMj supplementation to positively modulate the blood lipids profile following exercise. Previous studies in this field have only assessed the effect of POM supplementation on vascular function during different types of physical exercise [23–25, 67–69]. Indeed, POM supplementation has been reported to increase vessel diameter and blood flow, and to decrease systolic blood pressure and heart rate immediately following treadmill runs [67, 68], RSA test [69] and resistance exercises [63]. These beneficial effects of POMj have been linked to its abundant anti-oxidative polyphenolic compounds which are assumed to have cardioprotective effects [38, 70]. For example, POM polyphenols can protect LDL against cell-mediated oxidation by inhibiting macrophage lipid peroxidation [15, 39]. Similarly, POM polyphenols were shown to have the potential to activate endothelial nitric oxide synthase (eNOS) [70, 71], and to attenuate ROS-mediated nitric oxide scavenging [70], which is important given the vasodilatory properties of nitric [72]. Specifically, the higher post-exercise level of HDL-C following polyphenol-rich POMj consumption is likely due to the potential antioxidant effects conferred by its polyphenols content, leading to enhanced expression of genes related to HDL-C metabolism and function [73]. For example, by regulating cellular cholesterol efflux from macrophages and hepatic paraoxonase 1 expression and activity [74, 75]. In fact, previous clinical studies in a variety of human populations have shown that dietary polyphenol intake is associated with beneficial changes in serum biomarkers related to HDL function including increased HDL cholesterol concentration, as well as HDL antioxidant and cholesterol efflux capacities [75].

Taken together, these preliminary findings suggest a possible beneficial effect of dietary intake of natural polyphenol-rich POMj supplementation (250 ml POMj × 3 time daily) on some blood ion and lipid variables following high-intensity strength exercise in an athletic population. Although this modulation was limited to [Na^+^], [Cl^−^] and [HDL-C], it might have beneficial implications for resistance exercise performance and post exercise recovery among athletic population. Indeed, using similar dietary supplementation, we previously demonstrated that POMj improved weightlifting performance (+ 3.3% for the maximal lifted amount and + 8.3% for the total lifted amount) and attenuated the perception of muscle fatigue (− 4.4%) and knee extensor muscle soreness (13.4%) immediately and up to 48 h after a training session [25]. However, given that the resistance training session administered in the current study did not evoke significant ionic or lipid imbalance in the majority of the variables assessed, the improvement of resistance exercise performance previously shown in the same athletic population following POMj supplementation is unlikely to be due to improved maintenance of ionic or lipid balances, but more likely to other underlying mechanisms such as the attenuation of oxidative stress, muscle damage and inflammatory responses during and following exercise [23–25].

## Conclusion

This study is the first to test the efficacy of POMj supplementation to modulate plasma ion and lipid profiles following high-intensity resistance exercise in elite athletes. Plasma [K^+^] and [TG] were lower following resistance exercise compared to resting baseline values in the PLA condition, but not the POMj condition. Post-exercise plasma [Na^+^], [Cl^−^] and [HDL-C] were greater following POMj supplementation compared to PLA supplementation. Therefore, POMj supplementation has the potential to modulate some blood ionic and lipid responses to resistance exercise. However, given the small sample size, and given that only a small number of variables were modulated using POMj, further research in both athletic and non-athletic populations is needed to corroborate these preliminary observations and to elucidate the potential underlying mechanisms and translational potential of our novel observations in the current study.

### Ethics committee

Local committee of the Laboratory of Biochemistry, CHU Habib Bourguiba, Sfax University, Tunisia.

**Ethical registration number:** 16/2015.

## Data Availability

The datasets analyzed during the current study are available from the corresponding author on reasonable request.

## Abbreviations

ABCA1: ATP-binding cassette transporter A-1
Ca^2+^: Calcium
Cl^−^: Chloride
eNOS: Endothelial nitric oxide synthase
η^2^: Eta squared
1-RM: Heaviest weight lifted in a single repetition
HCT: Hematocrit
HB: Hemoglobin
HDL-C: High-density lipoprotein
HSD: Honest-significance-difference
HDL-C: Low-density lipoprotein
Mg^2+^: Magnesium
PLA: Placebo
POMj: Pomegranate juice
K^+^: Potassium
RBC: Red Blood Cells
RCT: Reverse cholesterol transport
Na^+^: Sodium
TC: Total cholesterol
TG: Triglyceride
